# Chromosome 4 Duplication Associated with Strabismus Leads to Gene Expression Changes in iPSC-Derived Cortical Neurons

**DOI:** 10.3390/genes16010080

**Published:** 2025-01-12

**Authors:** Mayra Martinez-Sanchez, William Skarnes, Ashish Jain, Sampath Vemula, Liang Sun, Shira Rockowitz, Mary C. Whitman

**Affiliations:** 1 Department of Ophthalmology, Boston Children’s Hospital, Boston, MA 02115, USA; mayra.martinezsanchez@childrens.harvard.edu (M.M.-S.); sampath.vemula@childrens.harvard.edu (S.V.); 2 Department of Ophthalmology, Harvard Medical School, Boston, MA 02115, USA; 3 Jackson Laboratory for Genomic Medicine, Farmington, CT 06032, USA; bill.skarnes@jax.org; 4 Research Computing, Department of Information Technology, Boston Children’s Hospital, Boston, MA 02115, USA; ashish.jain@childrens.harvard.edu (A.J.); liang.sun@childrens.harvard.edu (L.S.); shira.rockowitz@childrens.harvard.edu (S.R.); 5 Division of Genetics and Genomics, Manton Center for Orphan Disease Research, Boston Children’s Hospital, Boston, MA 02115, USA; 6 F.M. Kirby Neurobiology Center, Boston Children’s Hospital, Boston, MA 02115, USA

**Keywords:** strabismus, copy number variant, duplication, induced pluripotent stem cell, iPSC, genome editing, *SLITRK2*, protocadherin, *CSMD1*, *VGF*

## Abstract

Background/Objectives: Strabismus is the most common ocular disorder of childhood. Three rare, recurrent genetic duplications have been associated with both esotropia and exotropia, but the mechanisms by which they contribute to strabismus are unknown. This work aims to investigate the mechanisms of the smallest of the three, a 23 kb duplication on chromosome 4 (hg38|4:25,554,985-25,578,843). Methods: Using CRISPR and bridging oligos, we introduced the duplication into the Kolf2.1J iPSC line. We differentiated the parent line and the line with the duplication into cortical neurons using a three-dimensional differentiation protocol, and performed bulk RNASeq on neural progenitors (day 14) and differentiated neurons (day 63). Results: We successfully introduced the duplication into Kolf2.1J iPSCs by nucleofecting a bridging oligo for the newly formed junction along with cas9 ribonucleoparticles. We confirmed that the cells had a tandem duplication without inversion or deletion. The parent line and the line with the duplication both differentiated into neurons reliably. There were a total of 37 differentially expressed genes (DEGs) at day 63, 25 downregulated and 12 upregulated. There were 55 DEGs at day 14, 18 of which were also DEGs at day 63. The DEGs included a number of protocadherins, several genes involved in neuronal development, including *SLITRK2*, *CSMD1*, and *VGF*, and several genes of unknown function. Conclusions: A copy number variant (CNV) that confers risk for strabismus affects gene expression of several genes involved in neural development, highlighting that strabismus most likely results from abnormal neural development, and identifying several new genes and pathways for further research into the pathophysiology of strabismus.

## 1. Introduction

Strabismus, or a misalignment of the eyes, is the most common ocular disorder in children, and can lead to vision loss, amblyopia, and a loss of social and occupational opportunities [1,2,3,4]. Strabismus is classified by the alignment of the eyes relative to each other. The misalignment can be either horizontal or vertical. Horizontal misalignment can be divided into esotropia (eyes cross in) and exotropia (eyes deviate out). The pathophysiology of strabismus is not well understood. In certain rare disorders, the congenital cranial dysinnervation disorders (CCDDs), there are deficits of cranial motor neuron differentiation or axon guidance, which result in the inability to move one or both eyes fully, and lead to incomitant strabismus (strabismus that differs based on gaze position) [5]. However, the majority of strabismus patients can fully move both eyes and have concomitant strabismus (deviation is the same in all positions of gaze), with grossly normal development of the extraocular muscles [6,7,8,9].

Concomitant strabismus has a complex inheritance pattern. Although several studies have investigated strabismus as a Mendelian disorder, no causative genes have been identified, suggesting that strabismus may be oligogenic, meaning that multiple genes contribute to the inheritance, or polygenic [10]. Genome-wide association studies have identified only a few loci associated with strabismus [11,12]. We recently searched for copy number variants (CNVs) associated with strabismus and identified three rare, recurrent duplications that are significantly more common in both esotropia and exotropia patients than in controls [13,14]. Overall, one of these duplications is present in 10–11% of esotropia and exotropia patients [13,14]. The identified duplications are a 23 kb duplication on chromosome 4, a 464 kb duplication on chromosome 2 and a 344 kb duplication on chromosome 10, but the mechanisms by which the presence of any of these duplications increase the risk of strabismus remain unknown [13]. CNVs are deletions or duplications of large regions of the chromosome that can simultaneously disrupt multiple genes and regulatory regions, leading to changes in gene expression. In addition, CNVs have been shown to be important in several other neurodevelopmental disorders [15,16]. In this study, we investigate how this chromosome 4 duplication affects gene expression during neuronal development, starting from human induced pluripotent stem cells (iPSC). We chose to examine the 23 kb duplication first because it is much smaller and technically easier to introduce into iPSCs.

## 2. Materials and Methods

### 2.1. Introducing the 23 kb Chromosome 4 Duplication into iPSCs

We introduced the 23 kb chromosome 4 duplication (located at hg38: chr4:25554985-25578764) into the Kolf2.1J iPSC line [17] using CRISPR with a protocol to encourage homology-directed repair with bridging oligos [18]. We designed multiple guide RNAs to cut at the 5′ and 3′ sites of the duplication, tested their effectiveness in an in vitro assay, and chose the most effective guides. The final guides were CCAAACGTTCCGGCTTTGAACAA (5′ site) and GCTCGGTTCTCTGGTATAAATGG (3′ site). We designed bridging oligos for the 5′ site, new junction, and 3′ site. The oligos were as follows:

5′oligo_S_t, 5′AGGAAGGAGATGCTGTCTCTCTTCACTGTTCCTGCAATGCAGAACCAAACGTTCCGGCTTGAACAAAGGCATTGACCATAAGTTACTGGCTTGAGTATT;
Junction oligo, 5′-GGTTCCAGGACCCCCTGAAGATACCAAAATCCTGCTCGGTTCTCTGGTATGTTCCGGCTTTGAACAAAGGCATTGACCATAAGTTACTGGCTTGAGTATT
3′ oligo_S_t, 5′-AAAGTATACAGGAGAAAGTGCATGGGTTATATGCAAATACTATGCCATTTATACCAGAGAACCGAGCAGGATTTTGGTATCTTCAGGGGGTCCTGGAACC.

Briefly, iPS cells were grown in StemFlex media on Synthemax-coated wells. 2 × 10^5^ clonal iPS cells were plated in 3 mL StemFlex in each well of a Synthemax-coated 6-well plate. The media was changed every 2 days until cells were confluent. At 80% confluence, cells were passaged 1:15 using ReLeSR (STEMCELL Technologies Canada Inc., 05872, Vancouver, BC, Canada). The cells were nucleofected using an Amaxa 4D Nucleofector (Lonza) with the Primary Cell P3 program, pulse code CA-137. Prior to nucleofection, synthetic, modified sgRNAs (Synthego) were resuspended overnight at 4 °C in TE buffer at 4 µg/µL. Alt-R HDR modified ssODN (IDT) was resuspended at room temperature overnight in D-PBS at 200 pmol/µL; 4 µL sgRNA and 2 µL of recombinant Cas9 protein (20 µg HiFi Cas9 nuclease v3; IDT) were added to Primary P3 buffer (Lonza), and immediately prior to nucleofection, 1 µL (200 pmol) of Alt-R HDR modified ssODN was added. Then, 8 × 10^5^ cells were pelleted and 1 µL (200 pmol) of oligo repair template (either 5′, bridging, and 3′ or bridging only) was added to the Cas9 RNP, and cells were resuspended in 100 µL P3 solution and transferred to the vial containing Cas9RNP and oligo, transferred to the cuvette and nucleofected immediately. Cells were transferred to a synthemax-coated 6-well plate containing StemFlex +Revitacell media, and in half of the cases, HDR enhancer v2 (IDT) was added to the 1 µM final concentration. Cells were cultured at 32 °C, 5% CO_2_ for 2 days (cold shock). The media was replaced with StemFlex without Revitacell or HDR enhancer after 1 day. Once the cells reached 60–80% confluence, one well was dissociated with Accutase and 1500 single cells were seeded into a vitronectin-coated 10 cm dish in StemFlex with Revitacell. The media was replaced with StemFlex without Revitacell after 1 day. Ten days after plating single cells, individual colonies were picked and split into 2 corresponding 96-well plates—1 for freezing and 1 for lysing and genotyping of individual clones.

### 2.2. CRISPR Duplication Genotyping

Pooled genotyping was performed via PCR using primers for the new anticipated junction, and we found the strongest evidence of duplication formation using the bridging oligo alone, in the presence of HDR enhancer. The screening of 96 individual clones showed a single clone with the novel junctional sequence. This clone was further subcloned. Subclones were then genotyped by digital droplet PCR (ddPCR, BioRad, Hercules, CA, USA) [14] and showed a copy number of 3. PCR was used to ensure each clone had the new junction, the original 5′ and 3′ junctions (to ensure no inversion), and did not have the junction that would be created if a deletion occurred.

### 2.3. Whole Genome Sequencing

DNA from iPSCs with the duplication underwent whole-genome sequencing following human whole genome library preparation (350 bp) and sequencing using the NovaSeq X Plus series (PE150). The sequence was compared to the whole genome sequencing data of the parental Kolf line, which is publicly available at Hipsci.org. The presence of the duplication was confirmed and no other chromosomal rearrangements or single nucleotide variants were identified.

### 2.4. Cortical Neuron Generation and Characterization

Early passages from control (Kolf2.1J) human iPSCs and cells with the chromosome 4 duplication (before passage 10) were used to generate 3D cortical organoids by following an established protocol [19]. iPSC colonies at 85–90% confluence were dissociated into a single-cell suspension with Acutase solution; 5 × 10^6^ cells were plated in each well of low-cell-adhesion 6-well plates in a total of 5 mL of aggregation medium with 10 μM ROCK inhibitor and placed inside of an incubator on an orbital shaker. Then, 24 h after aggregation, the medium was replaced with induction medium containing neurobasal medium, DMEM/F12:Neurobasal (1:1), 1:100 N-2 supplement, 1:50 B27 supplement minus Vitamin A, 1% GlutaMAX supplement, 1:100 Pen/Strep, 1% MEM Non-Essential Amino Acids, 2-Mercaptoethanol (all from Thermo Fisher Scientific, Waltham, MA, USA) and L-Ascorbic Acid (Sigma, St. Louis, MO, USA), supplemented with 10 µM SB431542, 0.25 µM LDN193189, 5 µM XAV939 and 5 µM SU-5402 (added only in the first 24 h). On day 7, the medium was replaced with 5 mL of neurobasal medium containing 20 ng/mL EGF and 20 ng/mL FGF-2; the medium was then changed every other day. To promote growth of the 3D neural spheroids, media was changed at day 21 to differentiation basal medium DMEM/F12:Neurobasal (1:1), 1:100 N-2 supplement, 1:50 B27 supplement, 1% GlutaMAX supplement, 1:100 Pen/Strep, 1% MEM Non-Essential Amino Acids, and 2-Mercaptoethanol, supplemented with 20 ng/mL NGF, 20 ng/mL NT-3 and 20 ng/mL BDNF; the medium was changed every other day until day 42. Cortical neuron maturation medium (Neurobasal-A, B27 supplement, GlutaMAX supplement, Pen Strep and L-Ascorbic) was added at day 42 and replenished every 2–3 days until the end point for dissociation at day 63.

Cells were collected at day 14 (neural progenitor cells) and day 63 (differentiated cortical neurons) for characterization, and the remaining pellets were quick-frozen and stored at –80 °C for transcriptome analysis. Four independent differentiations were performed, each with both genotypes.

### 2.5. RNA Extraction and Library Preparation

Total RNA from both the control and cells with the duplication from each of the 4 independent differentiations was extracted from the pelleted cells using RNeasy Plus Mini Kit (Cat. No./ID: 74134, Qiagen, Germantown, MD, USA) by following the manufacturer’s instructions. The quantity of the extracted RNA was measured using a NanoDrop 8000 Spectrophotometer (Thermo Fisher Scientific Inc., Waltham, MA, USA) and integrity was assessed using an Agilent TapeStation 4200 system (Agilent Technologies, Santa Clara, CA, USA). Then, 200 ng of high-quality RNA from each sample was used for total RNA sequencing library preparation with the TruSeq Stranded Total RNA Library Prep Kit with Ribo-Zero Plus for Human/Mouse/Rat (Cat. No. RS-122-2201 (Illumina Inc., San Diego, CA, USA), following the manufacturer’s instructions. Final library concentration and quality was assessed using Qubit 3.0 Fluorometer (Thermo Fisher Scientific Inc., Waltham, MA, USA) and the Agilent TapeStation 4200 system (Agilent Technologies, Santa Clara, CA, USA). The libraries were normalized to 10 pM and sequenced on an Illumina NextSeq 550 System and NovaSeq PE150 platform.

### 2.6. RNA-Seq Data Analysis and Identification of Differentially Expressed Genes (DEGs)

We used trimmomatic (v0.39) [20] to trim the low-quality next-generation sequencing (NGS) reads (-threads 20 ILLUMINACLIP:TruSeq3-PE.fa:2:30:10 LEADING:3 TRAILING:3 SLIDINGWINDOW:4:15 MINLEN:36). Subsequently, only the high-quality trimmed reads were aligned to the human reference genome (hg38) using STAR (v2.7.2b) [21]. The reads counts were calculated by featureCounts software (v2.0.3) [22]. The expression heatmap used to show the log2 transformed expression (log2(FPKM + 1)) of the stem cell marker genes was generated using the pheatmap R package (v1.0.2) [23]. Differentially expressed genes (DEGs) were identified by using the DESeq2 R package (v1.38.3) (adjusted *p* value ≤ 0.05) [24]. We used the DiVenn tool (https://divenn.tch.harvard.edu/v2/) to compare the significantly differentially expressed genes between different conditions [25]. It visualizes the unique and common genes between selected comparisons in the form of networks. The upregulated and downregulated genes are marked as red and blue, respectively. The genes that are upregulated in one comparison and downregulated in others (or vice a versa) are marked as yellow. The expression heatmaps for the differentially expressed genes were generated using the pheatmap R package (v1.0.2) [23] by calculating the z-scores from the expression values.

### 2.7. RT-qPCR

1 μg of purified RNA, extracted using the RNeasy Plus Mini Kit, was retrotranscribed into cDNA using LunaScript RT SuperMix Kit (Cat. No. NEB #E3010, New England Biolabs, Ipswich, MA, USA). The PCR reaction was prepared with SsoAdvanced Universal SYBR Green Supermix (Cat. No. 1725270, Bio-Rad Laboratories, Hercules, CA, USA) and run on a CFX96 Dx Real-Time PCR Detection System (Cat. No. 1841000-IVD, Bio-Rad Laboratories). Three replications were performed for each sample, and the expression of each gene was normalized to *TBP*, which, based on the RNA-seq data, maintains a stable expression across genotypes and during differentiation.

### 2.8. Protein-Protein Interaction Network Analysis

The STRING database (Search Tool for the Retrieval of Interacting Genes/Proteins, https://string-db.org/) was used to construct a protein–protein interaction (PPI) network of the DEGs at day 63. Of the 12 DEGs, 9 with neuronal or synapse-related functions were identified in the database and included in the PPI network. Additional nodes were included in the network since no direct interactions were observed among the DEGs. An interaction score threshold of ≥0.4 was applied.

### 2.9. Immunocytochemical Staining

Spheroids were dissociated at day 63 with Papain Tissue Dissociation Kit according to the manufacturer’s recommended protocol (Worthington Biochemical), and 20,000 single cells were seeded into a Geltrex-coated 96-well plate. The cells were fixed in 4% paraformaldehyde for 20 min at room temperature and washed three times with DPBS. Fixed neurons were permeabilized with 0.4% Triton X-100 and 0.05% CHAPS in DPBS for 5 min, washed with DPBS twice, blocked in 0.1% Triton X-100 and 3% normal goat serum in DPBS for 30 min at room temperature, and incubated with chicken anti-MAP2 (1:1000, no. ab5392; Abcam, Cambridge, UK) and Synapsin 1 (1:250, no. AB1543P Millipore Sigma, St. Louis, MO, USA) overnight at 4 °C. Anti-Chicken IgY Alexa Fluor 488 and anti-rabbit IgG Alexa Fluor 647 were incubated for one hour at 37 °C. DAPI was used to stain for the nuclei for 10 min at room temperature.

## 3. Results

### 3.1. Model of Study

The chromosome 4 duplication with breakpoints at hg38 chr4:25,554,985 and chr4:25,578,843 includes exon 1 of the uncharacterized long non-coding RNA (lncRNA) *LOC101929161* (also known as *lnc-SEL1L3-2*), but no other genes. The duplication region identified in humans is only conserved in monkeys, and not in other mammals, and the lncRNA *LOC101929161* is present only in primates with no homology in mice. This duplication, therefore, cannot be easily modeled in an animal model, and we opted to study the effect of the duplication in a well-characterized human iPSC line [17] and perform cortical neuron differentiation to determine the effect of the duplication on neuronal differentiation.

### 3.2. Inserting the Duplication in iPSCs

**Figure 1:**
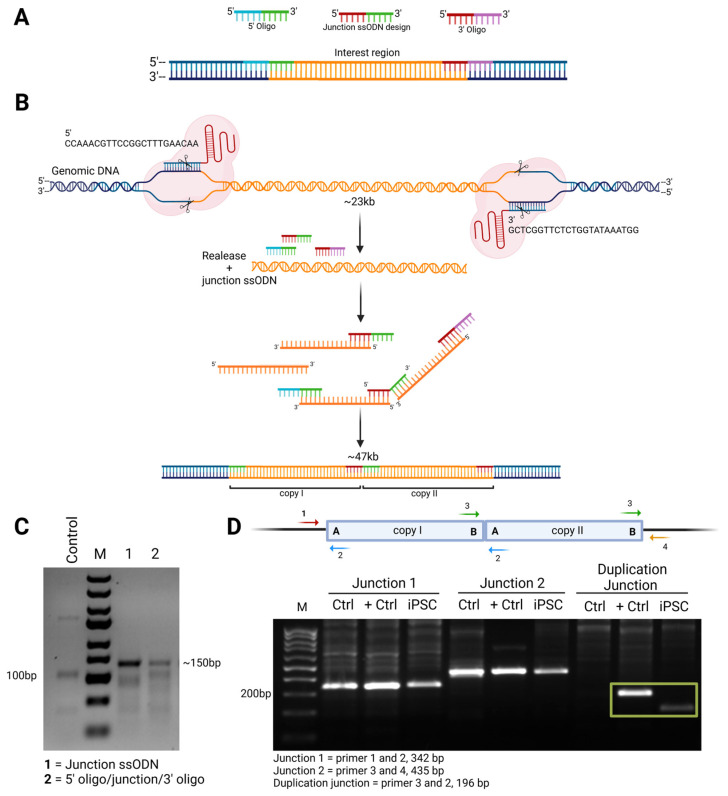
Schematic representation of genome editing to create the chromosome 4 duplication. (**A**) Representation for designed guide RNAs, bridging oligos for the 5′ site (blue section is the adaptor sequence and green section targets the 5’ end of the duplicated region), new desired junction site (red section targets the 3’ end of the duplicated region and green section targets the 5’ end of the duplicated region), and 3′ site (red section targets the 3’ end of the duplicated region and pink corresponds to adaptor sequence). (**B**) Schematic diagram of possible binding of the 5′ oligo, 3′ oligo and desired new junction, after which a new DNA strand is synthesized. Figure created with BioRender.com. (**C**) Pooled genotyping via PCR using primers for the new anticipated junction using junction ssODN oligo alone or 5′, junction and 3′ oligos. (**D**) End point PCR from control individual (no chromosome 4 duplication or strabismus, Ctrl), positive control (strabismic patient with presence of chromosome 4 duplication, +Ctrl), and selected edited iPSC sub-line (iPSC) testing for 5′ junction (Junction 1), 3′ junction (Junction 2) (to ensure no inversion) and new junction for duplication. The duplication junction in the iPSC line is slightly smaller than in the positive control individual because the iPSC duplication is 50 bp smaller than the originally identified duplication due to limitations in the design of the guide RNAs.

**Figure 2:**
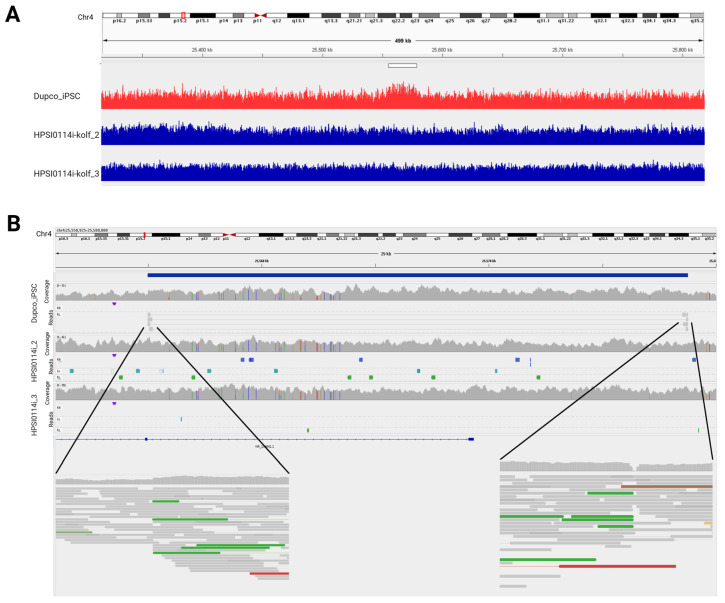
Whole-genome sequences from the new line with chromosome 4 duplication and parental line. (**A**) Read counts in chromosome 4, region of duplication in white bar, with a gain of read depth in the new line (red) compared with the parental line (blue). (**B**) Integrated Genome Viewer (IGV) views of sequencing reads from the chromosome 4 region of duplication (blue bar) from the new line (top and zoomed in below) and parental line (middle). In addition to the increased read depth, split reads are evident at the junctions (zoomed in below) that map to the newly created duplication junction.

To ensure a uniform genetic background, an early passage of the KOLF2.1J line [16] was used to insert the chromosome 4 duplication using a CRISPR technique with bridging oligos. The nucleofection method for the delivery of Cas9 RNP and ssODN was performed as described [18]. Since it is a 23 kb duplication, three different bridging oligos were designed flanking the 5′ site, new junction, and 3′ site (Figure 1A). Two guide RNAs were designed to cut at the 5′ and 3′ breakpoints (Figure 1B) and two different approaches were attempted to insert the duplication, one with the addition of the three bridging oligos and one including just the bridging oligo for the novel junction. Pooled genotyping via PCR for the new anticipated junction yielded the strongest evidence for the formation of the duplication using the bridging oligo alone (Figure 1C). After screening 96 individual clones, a single clone with the novel junctional sequence was found. This clone was further subcloned and six sub-lines were then genotyped by ddPCR, showing a copy number of 3. PCR was used to ensure the selected line had the new junction, the original 5′ and 3′ junctions (to ensure no inversion), and did not have the junction that would be created if a deletion occurred (Figure 1D). Due to limitations in the design of the guide RNAs, the resultant duplication is ~50 bp smaller than the duplication found in patients (Figure 1D). Once the presence of the duplication was confirmed, whole-genome sequencing was performed in the new line and compared with the parental line to ensure no other CNVs or SNVs were created during genome editing. Whole-genome sequencing showed a gain in read coverage in the duplicated region (Figure 2A), and split reads that correspond to the new junction were identified only in the edited line at the breakpoints of the duplication (Figure 2B). The analysis shows that the process of inserting the chromosome 4 duplication did not create any additional variants in the new line.

### 3.3. Differentiation into Cortical Neurons and RNA Library QC Measurements

**Figure 3:**
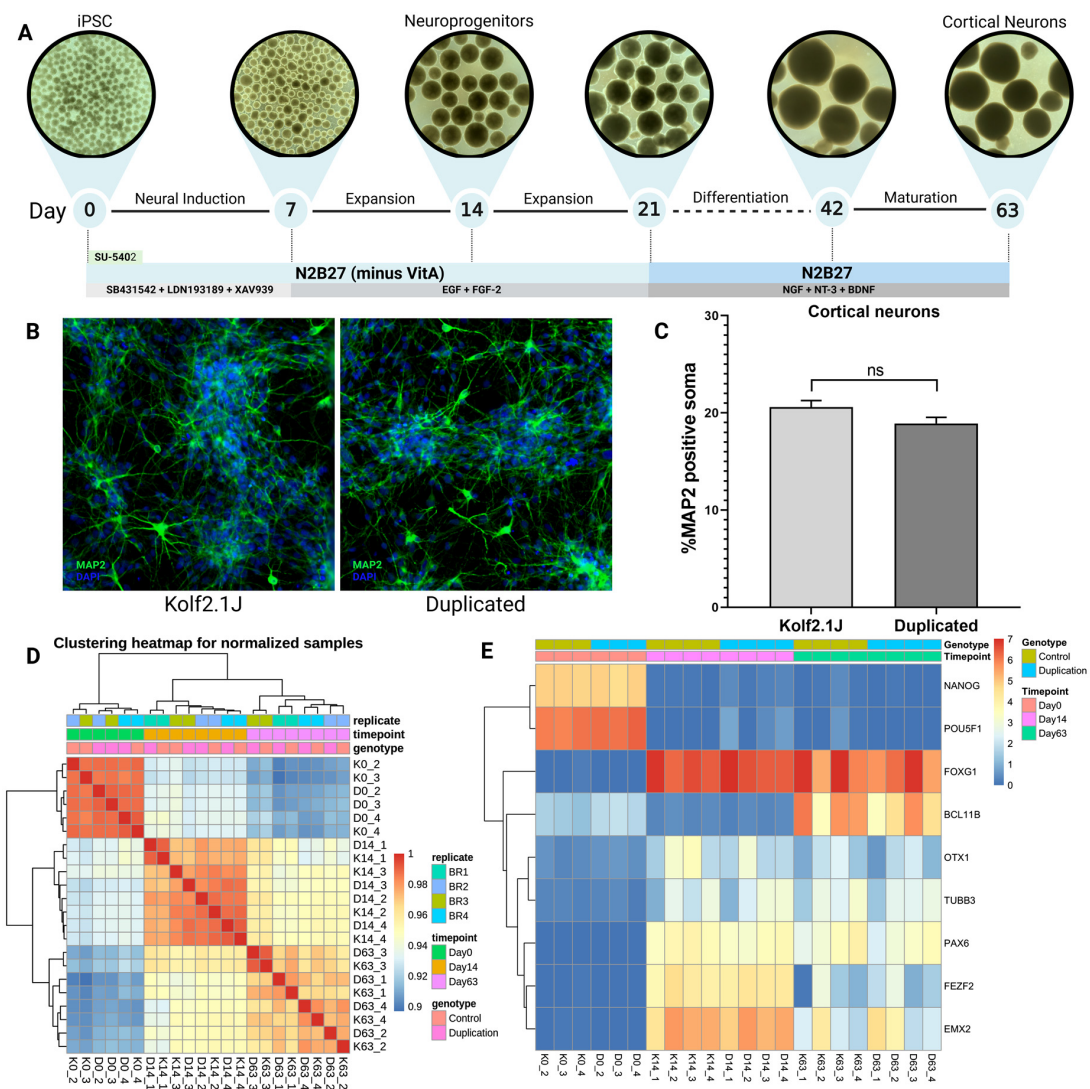
Schematic differentiation and validation of generated cortical neurons. (**A**) Schematic overview of the cortical neuron differentiation method, from day 0 to day 63. Figure created with BioRender.com. (**B**) Cortical neurons were dissociated at day 63 and immunocytochemical staining for MAP2 was performed 14 days after dissociation, in the control line (Kolf2.1J) and cells with chromosome 4 duplication (duplicated). (**C**) Quantification of MAP2 positive soma in control line (Kolf2.1J) and cells with duplication (duplicated); t-test was performed. (**D**) Quality control clustering heatmap from iPSCs, neuroprogenitor cells and cortical neurons at days 0 (three replicates), 14 and 63, respectively (four replicates), for control cells (K) and cells with duplication (**D**). (**E**) Heatmap for stem cell marker and neuronal marker expression using log2(FPKM + 1) values, comparing iPSCs (day 0), neuroprogenitor cells (day 14) and differentiated cortical neurons (day 63).

Control human iPSCs and cells with the chromosome 4 duplication were differentiated into cortical neurons using a protocol of dual Smad inhibition (SB431542 and LDN193189 SB) along with Wnt inhibition at the neural induction stage (Figure 3A) [19]. After the first week, the medium was supplemented with EGF and FGF-2 to promote expansion. Neuroprogenitor cells were assessed at day 14 for the expression of neuronal markers before proceeding with differentiation. From days 21 to 63, the differentiation and maturation of cortical neurons proceeded. Neurospheres were dissociated on day 63 and cultured in a monolayer. Neuronal identity and morphology were characterized through immunocytochemical staining for MAP2 and Synapsin1. Both lines reliably differentiated into neurons (Figure 3B,C).

To investigate transcriptional patterns during differentiation, RNA sequencing was conducted from four independent differentiations. As part of the sequencing quality control, samples were clustered by principal component analysis. Each time point clustered together (Figure 3D). We confirmed that neuroprogenitors and cortical neurons expressed neuronal markers and downregulated pluripotency genes (*NANOG* and *POU5F1*, expressed only at the iPSC stage), validating proper cortical differentiation (Figure 3E).

### 3.4. Identification of Differentially Expressed Genes (DEGs) Between Control and Cells with the Duplication

**Figure 4:**
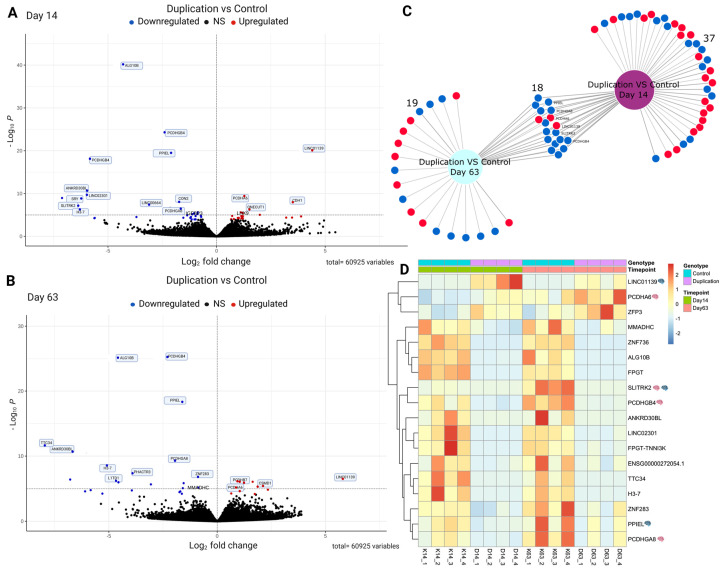
Differentially expressed genes in cells with the duplication. (**A**,**B**) Volcano plots showing the significant DEGs between control cells and cells with the duplication at the neuroprogenitor stage (**A**) and fully mature cortical neurons (**B**). Blue points represent downregulated genes, and red points represent upregulated genes. (**C**) DiVenn plot showing the unique and common DEGs for NPCs and cortical neurons. (**D**) Heatmap showing the expressions (z-scores) of shared DEGs at NPCs and cortical neuron stage from four replicates of control cells and cells with duplication.

To evaluate the effect of the duplication during neurodevelopment, we analyzed the gene expression profiles of control and duplicated cells. A total of 55 DEGs were identified at day 14, including 23 upregulated genes and 32 downregulated genes in cells with the duplication compared with the parental line (Figure 4A, Supplemental Table S1). Cortical neurons with the duplication (day 63) exhibited a total of 37 DEGs, including 12 upregulated and 25 downregulated genes, compared with the parental line, as shown in the volcano plot (Figure 4B, Supplemental Table S2). We found that 18 DEGs were shared between the neuroprogenitor stage and mature cortical neurons (Figure 4C), and their expression patterns are shown in the heatmap of Figure 4D. The lncRNA *LOC101929161* that overlaps the duplication was not differentially expressed and was expressed at very low levels in all samples. Several of the DEGs found at day 14 and day 63 have neuronal or synapse-related functions in the brain, or are genes associated with neurodevelopmental disorders (Table 1). Many of the identified genes are poorly annotated or have limited functional information, however, or have not been reported to have any function in the nervous system.

### 3.5. DEGs in Mature Cortical Neurons

**Figure 5:**
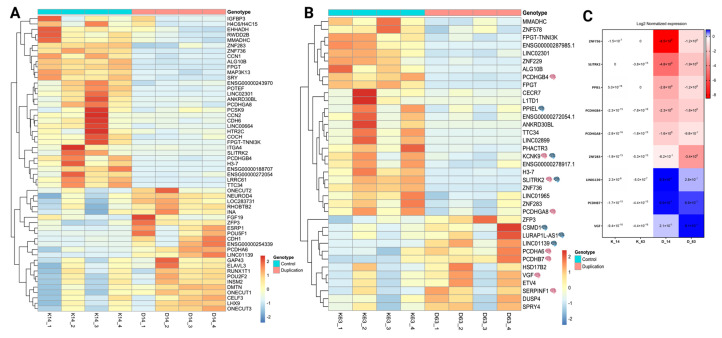
Heatmap of the DEGs in neuroprogenitors (**A**) and cortical neurons (**B**) and validation by qPCR (**C**). (**A**) Heatmap showing the expressions (z-scores) of differentially expressed genes in neuroprogenitor cells between controls and cells with duplication (fourth independent differentiations). (**B**) Heatmap showing the expressions (z-scores) of differentially expressed genes in cortical neurons between controls and cells with duplication, a pink brain next to the gene name represents a neuronal/synapse related function and a blue brain next to the gene name represents genes associated with neurodevelopmental disorders. (**C**) qRT-PCR validation from randomly selected upregulated and downregulated genes; total RNA from genotypes at day 14 and day 63 was used to analyze expression levels, and data were normalized using TBP as the reference gene.

**Figure 6:**
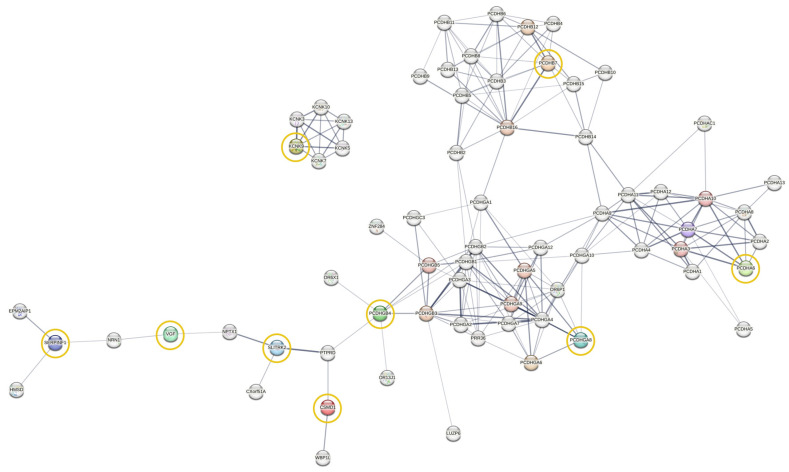
Protein–protein interaction (PPI) network of the DEGs in cortical neurons. The PPI interaction network of 9 relevant mapped neuronal function-related DEGs at day 63, constructed in the STRING database (DEGs highlighted in yellow circles).

To better characterize the changes in gene expression in neuroprogenitor cells and mature cortical neurons, we looked at the DEG patterns (Figure 5A,B). At day 63, several DEGs were genes with known functions in neuronal development and/or synapse formation (Figure 5B). These include the downregulation of *SLITRK2* and the upregulation of *SERPINF1* and *INA*, which are genes involved in synaptogenesis and synapse differentiation [40,41,42]. Four of the genes have been implicated in intellectual developmental disorders—*SLITRK2* [33,34], *KCNK9* [28], and *PPIEL* [31] are downregulated and *CSMD1* [26] is upregulated. Four members of the clustered protocadherins are differentially expressed, *PCDHGB4* and *PCDHGA8* are downregulated and *PCDHA6* and *PCDHB7* are upregulated. Protocadherin proteins play an important role in cell–cell interactions in neurons as well as pathway signaling [43,44,45]. Four zinc finger transcription factors (*ZNF578*, *ZNF229*, *ZNF736* and *ZNF283*) are differentially expressed, but the transcriptional targets are unknown for each of them. In order to corroborate these findings, qPCR was performed for several DEGs at days 14 and 63, and we confirmed the downregulation of *ZNF736*, *SLITRK2*, *PPIEL*, *PCDHGB4*, *PCDHGA8* and *ZNF283* and the upregulation of *LIN01139*, *PCDHB7* and *VGF* (Figure 5C). A protein–protein interaction network of DEGs at day 63 with neuron and synapse related functions was constructed using the STRING database to evaluate physical and functional associations (Figure 6). Although none of the DEGs directly interact with each other, many share interaction partners, such as SLITRK2, CSMD1, and PCDHB4, and all are associated with PTPRD. SLITRK2 and VGF are both associated with NPTX1.

## 4. Discussion

We successfully introduced a relatively large (23 kb) duplication associated with strabismus into human iPSCs, using CRISPR protocols designed to encourage homologous end-joining. Although the duplication occurred at low efficiency (~1%), it was technically feasible and did not create an inversion or deletion on the opposite chromosome. We show the differential expression of multiple genes during neurodevelopment when these cells are differentiated into cortical neurons, implicating several new pathways in strabismus pathophysiology.

Because this duplication is present in a region of the genome conserved only with primates, modeling the function of the duplication requires a human cell, rather than an animal model. Rather than create a line from an affected patient, we chose to genetically modify an existing, established iPSC line, so that cells with the duplication can be compared to otherwise genetically identical cells. The advances in genome engineering that encourage homologous end-joining made this possible. Similar methods should be able to create other structural variants in human iPSCs.

Cells from the parent and duplication line differentiated readily into neurons, showing that the duplication does not interfere with overall neuronal development. This is as expected, since patients with these duplications have isolated strabismus, without other neurological disorders. We thus expect subtle neuronal phenotypes.

Exon 1 of lncRNA *LOC101929161* is present in the duplicated region. We did not find the differential expression of this lncRNA, but it was expressed at very low levels in all samples. The expression level may be too low to be accurately captured using RNASeq, or we may not have captured an aberrant transcript if the duplication of exon 1 affects how this lncRNA is spliced.

We identified the differential expression of 74 genes in neural progenitors and/or neurons, 37 exclusively in neuroprogenitors, 18 in both neuroprogenitors and differentiated neurons, and 19 only in differentiated neurons. Of these, 14 have known functions in neuronal or synaptic development, which suggests that the duplication may increase the risk of strabismus via the dysregulation of genes involved in synapse formation or maintenance.

SLITRK2 is a transmembrane protein expressed in post-synaptic neurons. The six members of the Slit and Trk-like family regulate synapse development [46]. SLITRK2 is expressed in dendrites and triggers presynaptic differentiation in axons, and it also promotes excitatory synapse maintenance [46]. Missense variants in *SLITRK2* cause a neurodevelopmental disorder [34]; unfortunately, ocular phenotypes of the patients were not reported. The conditional knock-out of *Slitrk2* in mice caused a reduction in excitatory synapses [34]. We noted the downregulation of *SLITRK2* in cells with the strabismus-associated duplication, suggesting that a possible mechanism by which the duplication leads to strabismus is the downregulation of SLITRK2 and a resulting poor maintenance of excitatory synapses.

We noted the differential expression of several clustered protocadherins, with some upregulated and some downregulated. Clustered protocadherins are a family of cell-surface homophilic proteins that allow neurons to distinguish self from non-self [44]. Single nucleotide variants in protocadherins have been implicated in a variety of neurodevelopmental disorders, including autism and schizophrenia [44], which have high prevalences of strabismus [47,48,49]. The disruption of protocadherin expression levels in developing neurons may disrupt neurite repulsion and proper circuit formation, leading to strabismus.

*CSMD1 (CUB and Sushi Multiple Domains 1)* is upregulated in differentiated neurons with the duplication. CSMD1 is a transmembrane protein that regulates complement-mediated synapse elimination in the brain during development [50]. Intronic variants have been associated with schizophrenia via genome-wide association studies [51,52], and biallelic variants lead to a neurodevelopment disorder with intellectual disability, microcephaly, and polymicrogyria [26]. CSMD1 is involved in the complement-dependent synaptic refinement of retinogeniculate synapses in the lateral geniculate nucleus (LGN), a process that removes supernumerary retinal inputs during the first two postnatal weeks (in mice) and contributes to the segregation of the initially overlapping inputs from the two eyes [50]. A loss of *Csmd1* in mice leads to increased complement deposition, decreased retinogeniculate synapses, and a decreased area of binocular overlap in the LGN [48]. The over-expression of CSMD1 in humans may disrupt retinogeniculate synaptic refinement and the area of binocular overlap, contributing to strabismus.

VGF is a neurosecretory peptide that is processed into at least 12 VGF-derived peptides that have roles in neurogenesis, synaptogenesis, and learning and memory; VGF has been found to be dysregulated in multiple neurodegenerative and psychiatric disorders [39]. VGF is upregulated in neurons with the duplication. *SERPINF1* encodes Pigment Epithelial Derived Factor (PEDF), a protein with multiple functions, including retinal pigment epithelial function [53] and cortical neuromorphogenesis [54]. Protein–protein interaction network analysis shows that *SERPINF1*, *VGF*, *SLITRK2*, *CSMD1*, and *PCDHGB4* are all linked, although they do not interact directly.

*PPIEL* and *KCNK9* are both found in imprinted regions, and disruptions of imprinting are associated with neuropsychiatric disorders [29,31]. Several lncRNAs are differentially expressed, including LINC01139, which has been implicated in several cancers, including glioma [30]. NEUROD4 is a basic helix–loop–helix transcription factor important in neurogenesis [55] that is upregulated in neuroprogenitors with the duplication. Several other transcription factors are also dysregulated, including POU5F1, POU2F2, and several zinc finger transcription factors. Multiple other genes are differentially expressed, many of unknown function.

## 5. Conclusions

A genetic duplication that increases the risk of esotropia and exotropia causes gene expression changes in neuronal precursors and differentiated cortical neurons. Differentially expressed genes include several with known functions in neuronal and synapse development, including *SLITRK2*, *VGF*, *KCNK9*, and *CSMD1*, as well as several protocadherins, several transcription factors, and several genes of unknown function. These findings reinforce that strabismus is a neurodevelopmental disorder, and identify a number of pathways that may have roles in strabismus. Each of these genes is also now a candidate gene for further genetic studies of strabismus. The further investigation of the identified genes and molecular pathways will lead to further insights into strabismus pathophysiology, and may lead to new treatments.

## Figures and Tables

**Table 1:** Differentially expressed genes associated with brain function.

Gene	Description	Function	Diseases Associated
*CSMD1*	CUB and Sushi multiple domains 1	Component of membrane	Intellectual disability and schizophrenia [26,27]
*INA*	Internexin neuronal intermediate filament protein α	Morphogenesis of neurons	
*KCNK9*	Potassium two pore domain channel subfamily K member 9	Gene is imprinted in the brain	Birk–Barel Syndrome and Intellectual Disability [28,29]
*LINC01139*	Long intergenic non-protein coding RNA 1139	Unknown	Glial tumor [30]
*LURAP1L-AS1*	LURAP1L antisense RNA 1		Overlaps with TYRP1 gene associated with Albinism
*NEUROD4*	Neuronal differentiation 4	Mediates neuronal differentiation	
*PCDHA6*	Protocadherin α 6	Cell–cell connections in the brain	
*PCDHB7*	Protocadherin β 7	Cell–cell neural connections	
*PCDHGA8*	Protocadherin γ subfamily A, 8	Cell–cell connections in the brain	
*PCDHGB4*	Protocadherin γ subfamily B, 4	Cell–cell connections in the brain	
*PPIEL*	Peptidylprolyl isomerase E like pseudogene	Unknown	May be associated with intellectual disability and bipolar disorder [31,32]
*SERPINF1*	Serpin family F member 1	Neuronal differentiation in retinoblastoma cells	
*SLITRK2*	SLIT and NTRK like family member 2	Synaptogenesis and excitatory synapse differentiation	Intellectual developmental disorder, X-Linked 111, Retinitis Pigmentosa 6 and bipolar disorder [33,34,35,36].
*VGF*	VGF nerve growth factor inducible	Neurogenesis and neuroplasticity	Parkinson’s disease, Alzheimer’s, Huntington’s, front-temporal lobar dementia, pain, schizophrenia and depression [37,38,39]

## Data Availability

The original data presented in the study are openly available in dbGAP at pending.

## Supplementary Materials

The following supporting information can be downloaded at: https://www.mdpi.com/article/10.3390/genes16010080/s1, Table S1: DEGs at Day 14, Table S2: DEGs at Day 63, Table S3: Common DEGs at day 14 & 63.
