# Supplementary material for: Chromosome 4 Duplication Associated with Strabismus Leads to Gene Expression Changes in iPSC-Derived Cortical Neurons
Source: Genes (Basel). 2025 Jan 12;16(1):80. doi: 10.3390/genes16010080 (PMC11764630; doi:10.3390/genes16010080)
Supplement: Supplementary file 1 [file genes-16-00080-s001.zip › genes-3406351-supplementary.pdf]

Supplementary Table S1. DEGs at Day 14.

Gene	Chromosome	Description	Log2foldchange	Pvalue	Padj
**RHOBTB2**	Chromosome8	Rho related BTB domain containing 2	0.669612	9.83E-05	0.04719
**POU2F2**	Chromosome19	POU class 2 homeobox 2	1.155037	2.21E-05	0.01625
**CDH1**	Chromosome16	cadherin 1	3.513451	9.74E-09	1.74E-05
**MAP3K13**	Chromosome3	mitogen-activated protein kinase kinase kinase 13	-0.70969	2.88E-05	0.019962
**RUNX1T1**	Chromosome8	RUNX1 partner transcriptional co-repressor 1	1.025548	4.82E-05	0.027932
**PCDHA6**	Chromosome5	protocadherin alpha 6	1.279984	4.07E-10	1.02E-06
**COCH**	Chromosome14	cochlin	-0.96613	1.8E-05	0.014529
**ESRP1**	Chromosome8	epithelial splicing regulatory protein 1	3.205117	4.13E-05	0.026408
**CDH6**	Chromosome5	cadherin 6	-1.01866	0.000108	0.049112
**EHHADH**	Chromosome3	enoyl-CoA hydratase and 3-hydroxyacyl CoA dehydrogenase	-1.1781	2.84E-05	0.019962
**ITGA4**	Chromosome2	integrin subunit alpha 4	-1.20922	4.78E-05	0.027932
**CCN2**	Chromosome6	cellular communication network factor 2	-1.71999	8.53E-09	1.64E-05
**ONECUT2**	Chromosome18	one cut homeobox 2	0.717125	1.69E-05	0.014524
**NEUROD4**	Chromosome12	neuronal differentiation 4	1.181789	6.27E-05	0.03249
**LRRC61**	Chromosome7	leucine rich repeat containing 61	-5.62667	5.64E-05	0.030596
**CCN1**	Chromosome1	cellular communication network factor 1	-0.72065	1.48E-05	0.013234
**LHX9**	Chromosome1	LIM homeobox 9	1.20025	3.6E-06	0.004084
**IGFBP3**	Chromosome7	insulin like growth factor binding protein 3	-1.03333	3.92E-06	0.004255
**HTR2C**	ChromosomeX	5-hydroxytryptamine receptor 2C	-0.96333	0.000105	0.048926
**INA**	Chromosome10	internexin neuronal intermediate filament protein alpha	0.991458	4.99E-05	0.027932
**RWDD2B**	Chromosome21	RWD domain containing 2B	-1.14682	0.000106	0.048926
**DMTN**	Chromosome8	dematin actin binding protein	1.142605	6.28E-05	0.03249
**CELF3**	Chromosome1	CUGBP Elav-like family member 3	1.000989	9.04E-05	0.044241
**FGF19**	Chromosome11	fibroblast growth factor 19	0.976341	5.04E-05	0.027932
**ANKRD30BL**	Chromosome2	ankyrin repeat domain 30B like	-5.96489	1.92E-11	5.99E-08
**ZNF283**	Chromosome19	zinc finger protein 283	-0.87737	6.18E-06	0.006425
**MMADHC**	Chromosome2	metabolism of cobalamin associated D	-0.86747	2.33E-06	0.002912
**INSM2**	Chromosome14	INSM transcriptional repressor 2	1.211584	6.38E-05	0.03249
**PCSK9**	Chromosome1	proprotein convertase subtilisin/kexin type 9	-1.11235	7.03E-06	0.007017
**ONECUT1**	Chromosome15	one cut homeobox 1	1.516017	5.34E-07	0.000741
**GAP43**	Chromosome3	growth associated protein 43	1.213262	1.95E-05	0.015212
**ALG10B**	Chromosome12	ALG10 alpha-1,2-glucosyltransferase B	-4.29585	9.12E-41	2.28E-36
**ZFP3**	Chromosome17	ZFP3 zinc finger protein	1.120217	2.53E-06	0.003003
**SRY**	ChromosomeY	sex determining region Y	-6.22461	1.85E-09	3.86E-06
**SLITRK2**	ChromosomeX	SLIT and NTRK like family member 2	-6.35064	8.61E-08	0.000134
**ZBED10P**	Chromosome7	zinc finger BED-type containing 10, pseudogene	-5.6076	4.98E-05	0.027932
**ELAVL3**	Chromosome19	ELAV like RNA binding protein 3	0.793427	8.96E-05	0.044241
**POTEF**	Chromosome2	POTE ankyrin domain family member F	-3.68946	3E-05	0.020205
**POU5F1**	Chromosome6	POU class 5 homeobox 1	3.475177	4.08E-05	0.026408

**ONECUT3**	Chromosome19	one cut homeobox 3	1.989863	9.24E-06	0.008872
**LINC01139**	Chromosome1	long intergenic non-protein coding RNA 1139	4.400746	8.05E-21	5.02E-17
**TTC34**	Chromosome1	tetratricopeptide repeat domain 34	-9.11989	1.69E-17	7.04E-14
**ZNF736**	Chromosome7	zinc finger protein 736	-11.8101	6.25E-25	6.01E-21
**PPIEL**	Chromosome1	peptidylprolyl isomerase E like pseudogene	-2.09503	4.03E-20	2.01E-16
**UNKNOWN**	Chromosome15	novel transcript	1.138439	1.8E-05	0.014529
**PCDHGA8**	Chromosome5	protocadherin gamma subfamily A, 8	-1.66447	3.37E-07	0.000495
**PCDHGB4**	Chromosome5	protocadherin gamma subfamily B, 4	-2.41996	7.22E-25	6.01E-21
**UNKNOWN**	Chromosome8	novel transcript	3.890177	2.21E-05	0.01625
**FPGT**	Chromosome1	fucose-1-phosphate guanylyltransferase	-9.0428	3.81E-17	1.36E-13
**LINC02301**	Chromosome14	long intergenic non-protein coding RNA 2301	-5.96868	2.72E-10	7.55E-07
**FPGT-TNNI3K**	Chromosome1	FPGT-TNNI3K readthrough	-7.11343	1.10E-09	2.49E-06
**LINC00664**	Chromosome19	long intergenic non-protein coding RNA 664	-3.11982	4.42E-08	7.36E-05
**UNKNOWN**	Chromosome2	novel transcript	-1.53096	4.47E-05	0.027909
**H3-7**	Chromosome1	H3.7 histone (putative)	-6.27514	7.13E-07	0.000936
**H4C6**	Chromosome6	H4 clustered histone 6	-1.35358	1.22E-05	0.011257

Supplementary Table S2. DEGs at Day 63.

Gene	Chromosome	Description	Log2foldchange	Pvalue	Padj
**PCDHA6**	Chromosome5	protocadherin alpha 6	0.872531	6.41E-06	0.006875
**HSD17B2**	Chromosome16	hydroxysteroid 17-beta dehydrogenase 2	2.342497	1.39E-05	0.013431
**PHACTR3**	Chromosome20	phosphatase and actin regulator 3	-3.89591	4.05E-08	0.000106
**PCDHB7**	Chromosome5	protocadherin beta 7	1.038808	8.48E-07	0.001234
**DUSP4**	Chromosome8	dual specificity phosphatase 4	0.650908	5.28E-05	0.040641
**VGF**	Chromosome7	VGF nerve growth factor inducible	1.639064	8.36E-07	0.001234
**SERPINF1**	Chromosome17	serpin family F member 1	1.737584	6.36E-05	0.045024
**ANKRD30BL**	Chromosome2	ankyrin repeat domain 30B like	-6.66828	1.94E-11	6.33E-08
**ZNF283**	Chromosome19	zinc finger protein 283	-0.87006	1.47E-07	0.000349
**MMADHC**	Chromosome2	metabolism of cobalamin associated D	-0.87092	6.57E-06	0.006875
**KCNK9**	Chromosome8	potassium two pore domain channel subfamily K member 9	-1.5854	8.15E-06	0.008202
**ALG10B**	Chromosome12	ALG10 alpha-1,2-glucosyltransferase B	-4.57739	6.98E-26	9.13E-22
**ETV4**	Chromosome17	ETS variant transcription factor 4	1.039042	2.18E-05	0.018985
**ZFP3**	Chromosome17	ZFP3 zinc finger protein	1.245829	1.19E-06	0.001641
**CSMD1**	Chromosome8	CUB and Sushi multiple domains 1	2.122138	3.46E-06	0.004121
**SLITRK2**	ChromosomeX	SLIT and NTRK like family member 2	-10.4611	1.04E-15	6.78E-12
**SPRY4**	Chromosome5	sprouty RTK signaling antagonist 4	0.939426	6.72E-07	0.001173
**LINC01139**	Chromosome1	long intergenic non-protein coding RNA 1139	5.801755	2.93E-07	0.00064
**TTC34**	Chromosome1	tetratricopeptide repeat domain 34	-7.95605	2.19E-12	8.18E-09
**ZNF736**	Chromosome7	zinc finger protein 736	-8.6607	3.46E-15	1.81E-11
**LURAP1L-AS1**	Chromosome9	LURAP1L antisense RNA 1	1.872927	4.85E-06	0.005522
**CECR7**	Chromosome22	cat eye syndrome chromosome region, candidate 7	-3.05989	2.09E-06	0.00261
**L1TD1**	Chromosome1	LINE1 type transposase domain containing 1	-4.74316	4.77E-07	0.000891
**PPIEL**	Chromosome1	peptidylprolyl isomerase E like pseudogene	-1.60888	4.34E-19	3.78E-15
**LINC02899**	Chromosome5	long intergenic non-protein coding RNA 2899	-6.0976	2.48E-05	0.020682
**PCDHGA8**	Chromosome5	protocadherin gamma subfamily A, 8	-1.96081	5.30E-10	1.54E-06
**PCDHGB4**	Chromosome5	protocadherin gamma subfamily B, 4	-2.29778	6.18E-26	9.13E-22
**FPGT**	Chromosome1	fucose-1-phosphate guanylyltransferase	-8.73754	1.62E-14	7.09E-11
**LINC01965**	Chromosome2	long intergenic non-protein coding RNA 1965	-1.70088	2.53E-05	0.020682
**ZNF578**	Chromosome19	zinc finger protein 578	-1.74153	3.28E-05	0.02599
**LINC02301**	Chromosome14	long intergenic non-protein coding RNA 2301	-4.60798	7.23E-07	0.001183
**FPGT-TNNI3K**	Chromosome1	FPGT-TNNI3K readthrough	-3.93982	1.78E-05	0.016097
**UNKNOWN**	Chromosome2	novel transcript	-1.54812	1.36E-06	0.001783
**H3-7**	Chromosome1	H3.7 histone (putative)	-5.84017	1.62E-05	0.015106
**ZNF229**	Chromosome19	zinc finger protein 229	-5.2997	5.54E-05	0.041461
**UNKNOWN**	Chromosome19	novel transcript	-1.63122	5.79E-05	0.042081
**UNKNOWN**	Chromosome7	novel transcript	-6.78667	3.78E-07	0.00076

Supplementary Table S3. Common DEGs at day 14 & 63.

Gene	Chromosome	Function
**PCDHA6**	Chromosome5	Cell-cell connections in the brain
**ANKRD30BL**	Chromosome2	Unknown
**ZNF283**	Chromosome19	Transcriptional regulation
**MMADHC**	Chromosome2	Vitamin B12 metabolism
**ALG10B**	Chromosome12	Regulation of K+ channel, protein glycosylation
**ZFP3**	Chromosome17	DNA binding activity
**SLITRK2**	Chromosomex	Synaptogenesis and excitatory synapse differentiation
**LINC01139**	Chromosome1	Unknown
**TTC34**	Chromosome1	Unknown
**ZNF736**	Chromosome7	Transcriptional regulation
**PPIEL**	Chromosome1	Unknown pseudogene
**PCDHGA8**	Chromosome5	Cell-cell connections in the brain
**PCDHGB4**	Chromosome5	Cell-cell connections in the brain
**FPGT**	Chromosome1	Cell-cell recognition
**LINC02301**	Chromosome14	Unknown
**FPGT-TNNI3K**	Chromosome1	Unknown
**ENSG00000272054.1**	Chromosome2	Unknown
**H3-7**	Chromosome1	Unknown pseudogene
